# Benzyl *N*-(1-{*N*′-[(*E*)-2,3-dihy­droxy­benzyl­idene]hydrazinecarbon­yl}-2-hy­droxy­eth­yl)carbamate dihydrate

**DOI:** 10.1107/S1600536811029370

**Published:** 2011-07-30

**Authors:** Solange M. S. V. Wardell, Edward R. T. Tiekink, Marcus V. N. de Souza, Alessandra C. Pinheiro, James L. Wardell

**Affiliations:** a CHEMSOL, 1 Harcourt Road, Aberdeen AB15 5NY, Scotland; b Department of Chemistry, University of Malaya, 50603 Kuala Lumpur, Malaysia; c Fundação Oswaldo Cruz, Instituto de Tecnologia, em Fármacos – Farmanguinhos, R. Sizenando Nabuco, 100, Manguinhos, 21041-250 Rio de Janeiro, RJ, Brazil; d Centro de Desenvolvimento Tecnológico em Saúde (CDTS), Fundação Oswaldo Cruz (FIOCRUZ), Casa Amarela, Campus de Manguinhos, Av. Brasil 4365, 21040-900 Rio de Janeiro, RJ, Brazil

## Abstract

The organic mol­ecule in the title dihydrate, C_18_H_19_N_3_O_6_·2H_2_O, adopts a twisted U-shape with the major twists evident about the chiral C atom [the C—N—C—C torsion angle is −88.2 (4) °] and about the oxygen–benzyl bond [C—O—C—C = 74.2 (4) °]. The conformation about the imine bond [1.290 (4) Å] is *E* and an intra­molecular O—H⋯N hydrogen bond helps to establish the near coplanarity of the hy­droxy­benzene and hydrazine groups. The crystal packing features O—H⋯O and N—H⋯O hydrogen bonds, leading to two-dimensional supra­molecular arrays in the *ab* plane with weak C—H⋯π connections between the arrays.

## Related literature

For background to the use of l-serine derivatives in anti-tumour therapy, see: Jiao *et al.* (2009 ▶); Yakura *et al.* (2007 ▶). For background to *N*-acyl­hydrazone derivatives from l-serine for anti-tumour testing, see: de Souza *et al.* (2010 ▶, 2011 ▶); Pinheiro *et al.* (2010 ▶, 2011 ▶); Howie *et al.* (2011 ▶); Tiekink *et al.* (2011 ▶); Wardell *et al.* (2011 ▶).
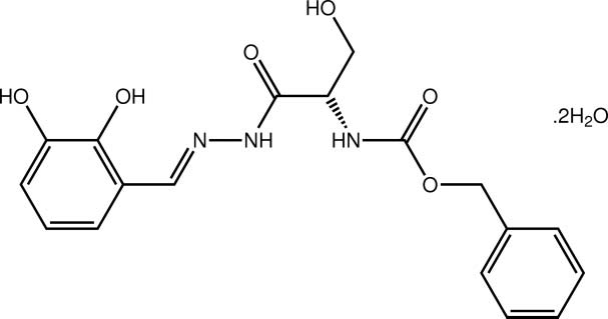


## Experimental

### 

#### Crystal data

C_18_H_19_N_3_O_6_·2H_2_O
*M* *_r_* = 409.39
Orthorhombic, 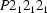

*a* = 4.7570 (2) Å
*b* = 13.1011 (4) Å
*c* = 30.5511 (9) Å
*V* = 1904.00 (11) Å^3^
*Z* = 4
Mo *K*α radiation
μ = 0.11 mm^−1^
*T* = 120 K
0.18 × 0.12 × 0.10 mm

#### Data collection

Bruker–Nonius Roper CCD camera on κ-goniostat diffractometer
Absorption correction: multi-scan (*SADABS*; Sheldrick, 2007 ▶) *T*_min_ = 0.887, *T*_max_ = 1.000
12958 measured reflections
2560 independent reflections
1984 reflections with *I* > 2σ(*I*)
*R*_int_ = 0.074

#### Refinement

*R*[*F* ^2^ > 2σ(*F* ^2^)] = 0.050
*wR*(*F* ^2^) = 0.114
*S* = 1.11
2560 reflections
289 parameters
9 restraints
H atoms treated by a mixture of independent and constrained refinement
Δρ_max_ = 0.23 e Å^−3^
Δρ_min_ = −0.26 e Å^−3^

### 

Data collection: *COLLECT* (Hooft, 1998 ▶); cell refinement: *DENZO* (Otwinowski & Minor, 1997 ▶) and *COLLECT*; data reduction: *DENZO* and *COLLECT*; program(s) used to solve structure: *SHELXS97* (Sheldrick, 2008 ▶); program(s) used to refine structure: *SHELXL97* (Sheldrick, 2008 ▶); molecular graphics: *ORTEP-3* (Farrugia, 1997 ▶) and *DIAMOND* (Brandenburg, 2006 ▶); software used to prepare material for publication: *publCIF* (Westrip, 2010 ▶).

## Supplementary Material

Crystal structure: contains datablock(s) global, I. DOI: 10.1107/S1600536811029370/hb6326sup1.cif

Structure factors: contains datablock(s) I. DOI: 10.1107/S1600536811029370/hb6326Isup2.hkl

Supplementary material file. DOI: 10.1107/S1600536811029370/hb6326Isup3.cml

Additional supplementary materials:  crystallographic information; 3D view; checkCIF report

## Figures and Tables

**Table 1:** Hydrogen-bond geometry (Å, °)

*D*—H⋯*A*	*D*—H	H⋯*A*	*D*⋯*A*	*D*—H⋯*A*
O1—H1o⋯N1	0.84 (2)	1.88 (2)	2.604 (3)	143 (3)
O2—H2o⋯O1w^i^	0.84 (2)	1.79 (2)	2.625 (3)	170 (3)
O4—H4o⋯O1^ii^	0.82 (3)	1.97 (3)	2.791 (3)	176 (3)
O1w—H1w⋯O2^iii^	0.85 (2)	2.05 (2)	2.894 (3)	169 (3)
O1w—H2w⋯O2w^ii^	0.84 (2)	2.04 (2)	2.879 (3)	176 (2)
O2w—H3w⋯O3	0.85 (2)	2.38 (2)	3.188 (3)	160 (3)
O2w—H4w⋯O3^iv^	0.86 (3)	1.97 (2)	2.818 (3)	168 (3)
N2—H2n⋯O5^iv^	0.87 (2)	2.08 (2)	2.892 (3)	154 (2)
N2—H2n⋯N3	0.87 (2)	2.34 (2)	2.705 (3)	106 (2)
N3—H3n⋯O2w^ii^	0.85 (3)	2.28 (3)	3.078 (3)	157 (3)
C18—H18⋯*Cg*1^v^	0.95	2.94	3.700 (3)	138

Symmetry codes: (i) 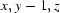; (ii) 
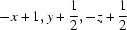
; (iii) 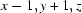; (iv) 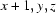; (v) 
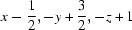
.

**Fig. 1.**
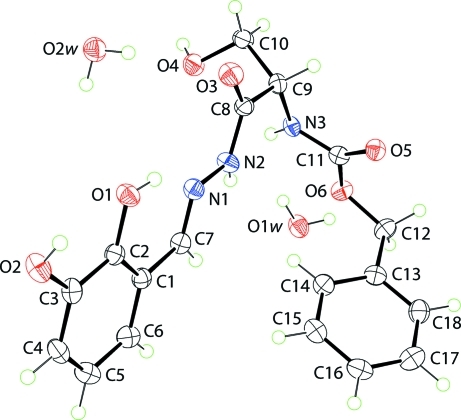
The molecular structure of (I) showing displacement ellipsoids at the 50% probability level.

**Fig. 2.**
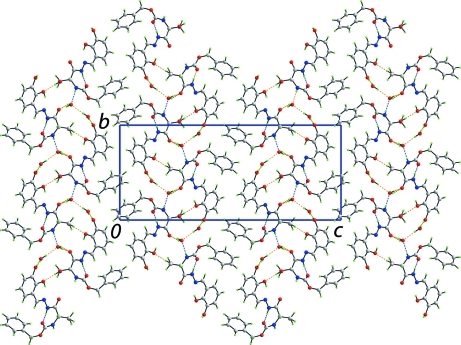
A view in projection down the a axis of the stacking of 2-D supramolecular arrays in the ab plane in (I), and with the O—H···O and N—H···O hydrogen bonding shown as orange and blue dashed lines, respectively.

## supplementary crystallographic information

### Comment

The motivation for investigations of molecules related to the title compound, (I), arise from the anti-tumour activity of *L*-serine derivatives (Jiao *et al.*, 2009; Yakura *et al.*, 2007) and, in particular, the development of *N*-acylhydrazone derivatives from *L*-serine for use in anti-tumour testing (Pinheiro *et al.*, 2010; de Souza *et al.*, 2010; Pinheiro *et al.*, 2011: Howie *et al.*, 2011; de Souza *et al.*, (2011); Howie *et al.* (2011); Tiekink *et al.* (2011); Wardell *et al.* (2011).

The crystallographic asymmetric unit of (I) comprises an organic molecule and two water molecules of crystallization. While the absolute structure could not be determined experimentally, the assignment of the *S*-configuration at the C9 atom is based on a starting reagent, *L*-serine. Overall, the organic molecule has a twisted U-shape with the two benzene rings lying to the same side of the molecule. Twists are evident about the chiral centre [the C11—N3—C9—C8 torsion angle is -88.2 (4) °] and about the benzyl group [C11—O6—C12—C13 is 74.2 (4) °]. The co-planarity at the hydroxylbenzene/hydrazine residue arises as a result of an intramolecular O1—H···N1 hydrogen bond (Table 1). The conformation about the N1═C7 imine bond [1.290 (4) Å] is *E*.

In the crystal, each of the acidic hydrogen atoms forms a significant hydrogen bond (Table 1). Thus, the O2- and O4-hydroxy groups form hydrogen bonds to the water-O1w and hydroxy-O1 atoms, respectively. The O1w-water molecule forms hydrogen bonds to the hydroxy-O2 and water-O2w atoms, while the O2w-water molecule forms connections to symmetry related carbonyl-O3 atoms, implying the latter is bifurcated. Finally, amine-N2—H is connected to carbonyl-O5, and amine-N3—H is connected to water-O2w. The result of the hydrogen bonding scheme is the formation of layers in the *ab* plane, Fig. 2. Connections between layers that stack along the *c* axis are of the form C—H···π, Table 1.

### Experimental

To a stirred solution of methyl (2*S*)-2-[(benzyloxycarbonyl)amino]-3-hydroxypropanoate (0.3 g, 1.17 mmol), prepared from (2*S*)-2-amino-3-hydroxypropanoate hydrochloride and benzyl chloroformate (21 ml, 0.15 mol), in ethanol (10 ml) was added N_2_H_4_.H2O (80%, 5.5 mmol). The reaction mixture was stirred for 24 h at room temperature, rotary evaporated and the residue washed with cold ethanol (3 *x* 10 ml) to give benzyl (1*S*)-2-hydrazino-1-(hydroxymethyl)-2-oxoethylcarbamate in 78% yield, which was used as such for the next stage. To a stirred solution of (*S*)-PhCH_2_OCONHCH(CH_2_OH)CONHNH_2_ (1.0 mmol) in ethanol (10 ml) at room temperature was added 2, 3-dihydroxybenzaldehyde (1.05 mmol). The reaction mixture was refluxed for 4 h, rotary evaporated and the residue purified by washing with cold ethanol (3 *x* 10 ml), affording the title compound, *M*.pt. 423 K, yield 81%. The sample for the structure determination was recrystallized from EtOH as pale-brown blocks of the dihydrate.  The water molecules were presumably absorbed from the atmosphere.  ^1^H NMR (500 MHz, DMSO-d6) δ (p.p.m.): 11.76 (1*H*, s, NHN), 10.95 (1*H*, s, C1—OH or C2—OH), 9.21 (1*H*, s, C1—OH or C2—OH), 8.41 (1*H*, s, N=CH),7.44 (1*H*, d, J= 7.4, NHCH), 7.40–7.20 (5*H*, m, Ph), 6.94 (1*H*, d, J= 7.8, H6), 6.85–6.80 (1*H*, m, H4), 6.73 (1*H*, t, J= 7.8, H5), 5.04 (3*H*, m, CH_2_Ph and OH), 4.15 (1*H*, m, CH), 3.75–3.55 (2*H*, m, CH_2_OH). ^13^C NMR (125 MHz, DMSO-d6) δ (p.p.m.): 171.1, 156.0, 145.6, 145.2, 141.6, 136.9, 128.4, 127.9, 127.8, 127.7, 120.0, 119.2, 117.4, 116.5, 65.4, 61.4, 56.3. IR (cm^-1^, KBr): 3270 (O—H), 1676 (COCH and COO). MS/ESI: [M—H]: 372.3.

### Refinement

The C-bound H atoms were geometrically placed (C–H = 0.95–1.00 Å) and refined as riding with *U*_iso_(H) = 1.2–1.5*U*_eq_(C). The O– and N-bound H atoms were located from a difference map and refined with the distance restraints O–H = 0.84 ± 0.01 and N–H = 0.86±0.01 Å, and with *U*_iso_(H) = *zU*_eq_(carrier atom); *z* = 1.5 for O and *z* = 1.2 for N. In the absence of significant anomalous scattering effects, 1782 Friedel pairs were averaged in the final refinement. However, the absolute configuration was assigned on the basis of the chirality of the *L*-serine starting material.

### Crystal data

**Table:**

C_18_H_19_N_3_O_6_·2H_2_O	*F*(000) = 864
*M**_r_* = 409.39	*D*_x_ = 1.428 Mg m^−^^3^
Orthorhombic, *P*2_1_2_1_2_1_	Mo *K*α radiation, λ = 0.71073 Å
Hall symbol: P 2ac 2ab	Cell parameters from 9601 reflections
*a* = 4.7570 (2) Å	θ = 2.9–27.5°
*b* = 13.1011 (4) Å	µ = 0.11 mm^−^^1^
*c* = 30.5511 (9) Å	*T* = 120 K
*V* = 1904.00 (11) Å^3^	Block, pale-brown
*Z* = 4	0.18 × 0.12 × 0.10 mm

### Data collection

**Table:**

Bruker–Nonius Roper CCD camera on κ-goniostat diffractometer	2560 independent reflections
Radiation source: Bruker–Nonius FR591 rotating anode	1984 reflections with *I* > 2σ(*I*)
graphite	*R*_int_ = 0.074
Detector resolution: 9.091 pixels mm^-1^	θ_max_ = 27.5°, θ_min_ = 3.1°
φ and ω scans	*h* = −6→5
Absorption correction: multi-scan (*SADABS*; Sheldrick, 2007)	*k* = −17→16
*T*_min_ = 0.887, *T*_max_ = 1.000	*l* = −37→39
12958 measured reflections	

### Refinement

**Table:**

Refinement on *F*^2^	Primary atom site location: structure-invariant direct methods
Least-squares matrix: full	Secondary atom site location: difference Fourier map
*R*[*F*^2^ > 2σ(*F*^2^)] = 0.050	Hydrogen site location: inferred from neighbouring sites
*wR*(*F*^2^) = 0.114	H atoms treated by a mixture of independent and constrained refinement
*S* = 1.11	*w* = 1/[σ^2^(*F*_o_^2^) + (0.034*P*)^2^ + 0.4778*P*] where *P* = (*F*_o_^2^ + 2*F*_c_^2^)/3
2560 reflections	(Δ/σ)_max_ < 0.001
289 parameters	Δρ_max_ = 0.23 e Å^−^^3^
9 restraints	Δρ_min_ = −0.26 e Å^−^^3^

### Special details

**Table:**

Geometry. All s.u.'s (except the s.u. in the dihedral angle between two l.s. planes) are estimated using the full covariance matrix. The cell s.u.'s are taken into account individually in the estimation of s.u.'s in distances, angles and torsion angles; correlations between s.u.'s in cell parameters are only used when they are defined by crystal symmetry. An approximate (isotropic) treatment of cell s.u.'s is used for estimating s.u.'s involving l.s. planes.
Refinement. Refinement of *F*^2^ against ALL reflections. The weighted *R*-factor *wR* and goodness of fit *S* are based on *F*^2^, conventional *R*-factors *R* are based on *F*, with *F* set to zero for negative *F*^2^. The threshold expression of *F*^2^ > 2σ(*F*^2^) is used only for calculating *R*-factors(gt) *etc*. and is not relevant to the choice of reflections for refinement. *R*-factors based on *F*^2^ are statistically about twice as large as those based on *F*, and *R*- factors based on ALL data will be even larger.

### Fractional atomic coordinates and isotropic or equivalent isotropic displacement parameters (Å^2^)

**Table:**

	*x*	*y*	*z*	*U*_iso_*/*U*_eq_	
O1	0.5296 (5)	0.14889 (15)	0.34576 (7)	0.0256 (5)	
H1O	0.435 (7)	0.2000 (19)	0.3378 (10)	0.038*	
O2	0.8776 (5)	0.00115 (16)	0.38088 (8)	0.0289 (6)	
H2O	0.719 (4)	−0.022 (3)	0.3735 (12)	0.043*	
O3	0.0260 (5)	0.33623 (15)	0.27559 (7)	0.0288 (6)	
O4	0.3510 (5)	0.54140 (18)	0.23084 (7)	0.0293 (6)	
H4O	0.391 (10)	0.571 (3)	0.2077 (11)	0.044*	
O5	−0.3437 (5)	0.58789 (16)	0.34957 (7)	0.0246 (5)	
O6	−0.0248 (5)	0.71487 (15)	0.35850 (6)	0.0249 (6)	
N1	0.4021 (7)	0.34210 (18)	0.34263 (8)	0.0222 (6)	
N2	0.2671 (6)	0.42717 (19)	0.32640 (8)	0.0225 (6)	
H2N	0.337 (7)	0.4863 (14)	0.3341 (10)	0.027*	
N3	0.0278 (6)	0.60659 (19)	0.30326 (8)	0.0218 (6)	
H3N	0.172 (9)	0.642 (2)	0.2984 (10)	0.026*	
C1	0.7354 (7)	0.2756 (2)	0.39369 (9)	0.0208 (7)	
C2	0.7133 (7)	0.1748 (2)	0.37819 (10)	0.0204 (7)	
C3	0.8820 (8)	0.0997 (2)	0.39619 (10)	0.0230 (7)	
C4	1.0684 (8)	0.1236 (2)	0.42946 (10)	0.0264 (8)	
H4	1.1846	0.0716	0.4414	0.032*	
C5	1.0887 (9)	0.2223 (2)	0.44573 (10)	0.0283 (8)	
H5	1.2161	0.2376	0.4688	0.034*	
C6	0.9210 (8)	0.2979 (2)	0.42797 (9)	0.0257 (8)	
H6	0.9318	0.3655	0.4391	0.031*	
C7	0.5738 (8)	0.3584 (2)	0.37454 (9)	0.0228 (7)	
H7	0.5949	0.4257	0.3857	0.027*	
C8	0.0844 (8)	0.4187 (2)	0.29279 (9)	0.0213 (7)	
C9	−0.0437 (8)	0.5184 (2)	0.27644 (9)	0.0213 (7)	
H9	−0.2529	0.5109	0.2763	0.026*	
C10	0.0533 (8)	0.5356 (3)	0.22979 (10)	0.0253 (8)	
H10A	−0.0080	0.4785	0.2109	0.030*	
H10B	−0.0269	0.5998	0.2181	0.030*	
C11	−0.1316 (8)	0.6322 (2)	0.33806 (10)	0.0213 (7)	
C12	−0.1522 (9)	0.7377 (2)	0.40029 (9)	0.0267 (8)	
H12A	−0.3592	0.7348	0.3973	0.032*	
H12B	−0.1009	0.8081	0.4090	0.032*	
C13	−0.0615 (8)	0.6646 (2)	0.43599 (10)	0.0240 (7)	
C14	0.1605 (8)	0.5972 (2)	0.43100 (10)	0.0286 (8)	
H14	0.2612	0.5948	0.4042	0.034*	
C15	0.2360 (8)	0.5330 (3)	0.46517 (10)	0.0305 (8)	
H15	0.3859	0.4859	0.4613	0.037*	
C16	0.0959 (9)	0.5366 (3)	0.50491 (10)	0.0299 (8)	
H16	0.1499	0.4931	0.5283	0.036*	
C17	−0.1231 (9)	0.6044 (3)	0.50978 (11)	0.0342 (9)	
H17	−0.2201	0.6079	0.5369	0.041*	
C18	−0.2039 (8)	0.6676 (3)	0.47569 (10)	0.0295 (8)	
H18	−0.3573	0.7132	0.4795	0.035*	
O1W	0.4103 (6)	0.91059 (18)	0.35680 (8)	0.0343 (6)	
H1W	0.248 (4)	0.936 (3)	0.3598 (11)	0.051*	
H2W	0.416 (8)	0.876 (2)	0.3336 (7)	0.051*	
O2W	0.5751 (6)	0.28389 (19)	0.22018 (7)	0.0310 (6)	
H3W	0.420 (4)	0.282 (3)	0.2339 (10)	0.047*	
H4W	0.695 (6)	0.303 (3)	0.2392 (9)	0.047*	

### Atomic displacement parameters (Å^2^)

**Table:**

	*U*^11^	*U*^22^	*U*^33^	*U*^12^	*U*^13^	*U*^23^
O1	0.0249 (15)	0.0216 (11)	0.0302 (12)	0.0024 (10)	−0.0065 (11)	−0.0023 (10)
O2	0.0241 (14)	0.0196 (12)	0.0430 (13)	−0.0005 (12)	−0.0046 (12)	0.0010 (10)
O3	0.0296 (15)	0.0218 (11)	0.0350 (12)	−0.0035 (12)	−0.0039 (12)	−0.0053 (10)
O4	0.0240 (14)	0.0372 (14)	0.0268 (12)	−0.0026 (12)	0.0030 (12)	0.0055 (11)
O5	0.0228 (14)	0.0230 (11)	0.0280 (11)	−0.0033 (11)	0.0013 (11)	−0.0011 (10)
O6	0.0295 (15)	0.0199 (11)	0.0254 (11)	−0.0061 (11)	0.0002 (11)	−0.0010 (9)
N1	0.0241 (16)	0.0187 (13)	0.0237 (13)	0.0026 (13)	−0.0005 (13)	0.0024 (11)
N2	0.0248 (17)	0.0155 (13)	0.0273 (13)	0.0008 (13)	−0.0066 (13)	0.0003 (11)
N3	0.0163 (16)	0.0215 (14)	0.0276 (14)	−0.0010 (13)	−0.0002 (13)	0.0001 (11)
C1	0.0201 (18)	0.0213 (16)	0.0210 (14)	−0.0004 (15)	0.0020 (15)	0.0014 (13)
C2	0.0199 (18)	0.0199 (16)	0.0214 (15)	−0.0011 (15)	0.0019 (15)	−0.0001 (13)
C3	0.0212 (19)	0.0184 (15)	0.0293 (16)	0.0006 (15)	0.0038 (15)	0.0021 (13)
C4	0.0189 (19)	0.0291 (17)	0.0314 (17)	0.0024 (16)	−0.0020 (16)	0.0060 (14)
C5	0.027 (2)	0.0305 (17)	0.0274 (16)	−0.0039 (18)	−0.0052 (16)	0.0053 (14)
C6	0.026 (2)	0.0254 (16)	0.0255 (15)	−0.0048 (17)	0.0005 (16)	0.0014 (14)
C7	0.0238 (19)	0.0212 (16)	0.0235 (15)	0.0002 (15)	0.0015 (16)	0.0008 (13)
C8	0.0195 (18)	0.0248 (16)	0.0195 (14)	−0.0026 (16)	0.0002 (14)	−0.0012 (13)
C9	0.0191 (18)	0.0201 (14)	0.0248 (15)	0.0008 (15)	0.0003 (15)	−0.0009 (12)
C10	0.023 (2)	0.0295 (17)	0.0232 (15)	−0.0018 (16)	−0.0027 (16)	0.0013 (14)
C11	0.024 (2)	0.0158 (14)	0.0240 (16)	−0.0003 (15)	−0.0037 (15)	0.0018 (12)
C12	0.034 (2)	0.0211 (16)	0.0247 (15)	−0.0012 (16)	0.0032 (16)	−0.0017 (13)
C13	0.0264 (19)	0.0186 (15)	0.0269 (16)	−0.0055 (16)	−0.0033 (15)	−0.0019 (13)
C14	0.026 (2)	0.0315 (18)	0.0279 (17)	−0.0005 (17)	−0.0006 (16)	−0.0013 (15)
C15	0.026 (2)	0.0289 (18)	0.0366 (19)	0.0015 (17)	−0.0031 (17)	0.0013 (16)
C16	0.030 (2)	0.0300 (18)	0.0299 (17)	−0.0053 (18)	−0.0082 (18)	0.0059 (15)
C17	0.042 (2)	0.0332 (19)	0.0276 (17)	−0.003 (2)	0.0038 (18)	−0.0001 (15)
C18	0.029 (2)	0.0257 (17)	0.0337 (18)	0.0031 (17)	0.0026 (17)	−0.0021 (15)
O1W	0.0290 (15)	0.0288 (13)	0.0451 (14)	0.0034 (13)	−0.0043 (13)	−0.0095 (11)
O2W	0.0283 (15)	0.0302 (12)	0.0346 (13)	0.0016 (14)	−0.0016 (12)	−0.0039 (11)

### Geometric parameters (Å, °)

**Table:**

O1—C2	1.364 (4)	C6—H6	0.9500
O1—H1O	0.842 (10)	C7—H7	0.9500
O2—C3	1.374 (4)	C8—C9	1.526 (4)
O2—H2O	0.843 (10)	C9—C10	1.515 (4)
O3—C8	1.233 (3)	C9—H9	1.0000
O4—C10	1.418 (4)	C10—H10A	0.9900
O4—H4O	0.83 (4)	C10—H10B	0.9900
O5—C11	1.216 (4)	C12—C13	1.515 (4)
O6—C11	1.350 (4)	C12—H12A	0.9900
O6—C12	1.445 (3)	C12—H12B	0.9900
N1—C7	1.290 (4)	C13—C14	1.385 (5)
N1—N2	1.379 (3)	C13—C18	1.390 (4)
N2—C8	1.350 (4)	C14—C15	1.388 (4)
N2—H2N	0.875 (10)	C14—H14	0.9500
N3—C11	1.349 (4)	C15—C16	1.386 (5)
N3—C9	1.456 (4)	C15—H15	0.9500
N3—H3N	0.84 (4)	C16—C17	1.378 (5)
C1—C6	1.401 (4)	C16—H16	0.9500
C1—C2	1.407 (4)	C17—C18	1.385 (5)
C1—C7	1.453 (4)	C17—H17	0.9500
C2—C3	1.383 (4)	C18—H18	0.9500
C3—C4	1.385 (5)	O1W—H1W	0.851 (10)
C4—C5	1.388 (4)	O1W—H2W	0.845 (10)
C4—H4	0.9500	O2W—H3W	0.848 (10)
C5—C6	1.383 (5)	O2W—H4W	0.852 (10)
C5—H5	0.9500		
			
C2—O1—H1O	111 (3)	N3—C9—H9	108.3
C3—O2—H2O	116 (3)	C10—C9—H9	108.3
C10—O4—H4O	104 (3)	C8—C9—H9	108.3
C11—O6—C12	114.7 (3)	O4—C10—C9	106.9 (3)
C7—N1—N2	115.6 (2)	O4—C10—H10A	110.3
C8—N2—N1	120.5 (2)	C9—C10—H10A	110.3
C8—N2—H2N	121 (2)	O4—C10—H10B	110.3
N1—N2—H2N	116 (2)	C9—C10—H10B	110.3
C11—N3—C9	120.6 (3)	H10A—C10—H10B	108.6
C11—N3—H3N	118 (2)	O5—C11—N3	125.2 (3)
C9—N3—H3N	122 (2)	O5—C11—O6	124.2 (3)
C6—C1—C2	119.6 (3)	N3—C11—O6	110.7 (3)
C6—C1—C7	118.6 (3)	O6—C12—C13	112.7 (3)
C2—C1—C7	121.8 (3)	O6—C12—H12A	109.1
O1—C2—C3	118.9 (3)	C13—C12—H12A	109.1
O1—C2—C1	121.7 (3)	O6—C12—H12B	109.1
C3—C2—C1	119.4 (3)	C13—C12—H12B	109.1
O2—C3—C4	118.2 (3)	H12A—C12—H12B	107.8
O2—C3—C2	121.6 (3)	C14—C13—C18	119.1 (3)
C4—C3—C2	120.2 (3)	C14—C13—C12	122.8 (3)
C3—C4—C5	121.2 (3)	C18—C13—C12	118.1 (3)
C3—C4—H4	119.4	C13—C14—C15	120.1 (3)
C5—C4—H4	119.4	C13—C14—H14	120.0
C6—C5—C4	119.1 (3)	C15—C14—H14	120.0
C6—C5—H5	120.4	C16—C15—C14	120.9 (3)
C4—C5—H5	120.4	C16—C15—H15	119.5
C5—C6—C1	120.5 (3)	C14—C15—H15	119.5
C5—C6—H6	119.7	C17—C16—C15	118.7 (3)
C1—C6—H6	119.7	C17—C16—H16	120.6
N1—C7—C1	121.0 (3)	C15—C16—H16	120.6
N1—C7—H7	119.5	C16—C17—C18	121.0 (3)
C1—C7—H7	119.5	C16—C17—H17	119.5
O3—C8—N2	122.8 (3)	C18—C17—H17	119.5
O3—C8—C9	121.4 (3)	C17—C18—C13	120.3 (3)
N2—C8—C9	115.8 (3)	C17—C18—H18	119.9
N3—C9—C10	109.9 (3)	C13—C18—H18	119.9
N3—C9—C8	113.7 (2)	H1W—O1W—H2W	109 (2)
C10—C9—C8	108.3 (3)	H3W—O2W—H4W	105 (2)
			
C7—N1—N2—C8	179.6 (3)	O3—C8—C9—N3	174.4 (3)
C6—C1—C2—O1	178.0 (3)	N2—C8—C9—N3	−6.7 (4)
C7—C1—C2—O1	−3.1 (5)	O3—C8—C9—C10	−63.2 (4)
C6—C1—C2—C3	−2.0 (5)	N2—C8—C9—C10	115.7 (3)
C7—C1—C2—C3	176.9 (3)	N3—C9—C10—O4	64.5 (3)
O1—C2—C3—O2	3.0 (5)	C8—C9—C10—O4	−60.2 (3)
C1—C2—C3—O2	−177.0 (3)	C9—N3—C11—O5	−1.4 (5)
O1—C2—C3—C4	−179.4 (3)	C9—N3—C11—O6	179.0 (3)
C1—C2—C3—C4	0.7 (5)	C12—O6—C11—O5	10.5 (4)
O2—C3—C4—C5	178.4 (3)	C12—O6—C11—N3	−169.9 (3)
C2—C3—C4—C5	0.7 (5)	C11—O6—C12—C13	74.2 (4)
C3—C4—C5—C6	−0.6 (6)	O6—C12—C13—C14	12.3 (5)
C4—C5—C6—C1	−0.7 (5)	O6—C12—C13—C18	−169.4 (3)
C2—C1—C6—C5	2.0 (5)	C18—C13—C14—C15	0.8 (5)
C7—C1—C6—C5	−176.9 (3)	C12—C13—C14—C15	179.1 (3)
N2—N1—C7—C1	−178.2 (3)	C13—C14—C15—C16	−1.4 (5)
C6—C1—C7—N1	179.3 (3)	C14—C15—C16—C17	0.8 (5)
C2—C1—C7—N1	0.4 (5)	C15—C16—C17—C18	0.4 (5)
N1—N2—C8—O3	1.2 (5)	C16—C17—C18—C13	−1.0 (5)
N1—N2—C8—C9	−177.7 (3)	C14—C13—C18—C17	0.4 (5)
C11—N3—C9—C10	150.3 (3)	C12—C13—C18—C17	−178.0 (3)
C11—N3—C9—C8	−88.2 (4)		

### Hydrogen-bond geometry (Å, °)

**Table:**

*D*—H···*A*	*D*—H	H···*A*	*D*···*A*	*D*—H···*A*
O1—H1o···N1	0.84 (2)	1.88 (2)	2.604 (3)	143 (3)
O2—H2o···O1w^i^	0.84 (2)	1.79 (2)	2.625 (3)	170 (3)
O4—H4o···O1^ii^	0.82 (3)	1.97 (3)	2.791 (3)	176 (3)
O1w—H1w···O2^iii^	0.85 (2)	2.05 (2)	2.894 (3)	169 (3)
O1w—H2w···O2w^ii^	0.84 (2)	2.04 (2)	2.879 (3)	176 (2)
O2w—H3w···O3	0.85 (2)	2.38 (2)	3.188 (3)	160 (3)
O2w—H4w···O3^iv^	0.86 (3)	1.97 (2)	2.818 (3)	168 (3)
N2—H2n···O5^iv^	0.874 (19)	2.08 (2)	2.892 (3)	154 (2)
N2—H2n···N3	0.874 (19)	2.34 (2)	2.705 (3)	106 (2)
N3—H3n···O2w^ii^	0.85 (3)	2.28 (3)	3.078 (3)	157 (3)
C18—H18···Cg1^v^	0.95	2.94	3.700 (3)	138

Symmetry codes: (i) *x*, *y*−1, *z*; (ii) −*x*+1, *y*+1/2, −*z*+1/2; (iii) *x*−1, *y*+1, *z*; (iv) *x*+1, *y*, *z*; (v) *x*−1/2, −*y*+3/2, −*z*+1.

## References

[bb1] Brandenburg, K. (2006). *DIAMOND* Crystal Impact GbR, Bonn, Germany.

[bb2] Farrugia, L. J. (1997). *J. Appl. Cryst.* **30**, 565.

[bb3] Hooft, R. W. W. (1998). *COLLECT* Nonius BV, Delft, The Netherlands.

[bb4] Howie, R. A., de Souza, M. V. N., Pinheiro, A. C., Kaiser, C. R., Wardell, J. L. & Wardell, S. M. S. V. (2011). *Z. Kristallogr.* **226**, 483–491.

[bb5] Jiao, X., Wang, L., Xiao, Q., Xie, P. & Liang, X. (2009). *J. Asian Nat. Prod. Res.* **11**, 274–280. 10.1080/10286020902828369 19408153

[bb6] Otwinowski, Z. & Minor, W. (1997). *Methods in Enzymology*, Vol. 276, *Macromolecular Crystallography*, Part A, edited by C. W. Carter Jr & R. M. Sweet, pp. 307–326. New York: Academic Press.

[bb7] Pinheiro, A. C., Souza, M. V. N. de, Tiekink, E. R. T., Wardell, J. L. & Wardell, S. M. S. V. (2010). *Acta Cryst.* E**66**, o1004–o1005. 10.1107/S1600536810011463 PMC2983774 21580568

[bb8] Pinheiro, A. C., Souza, M. V. N. de, Tiekink, E. R. T., Wardell, S. M. S. V. & Wardell, J. L. (2011). *Acta Cryst.* E**67**, o581–o582. 10.1107/S1600536811003795 PMC3052090 21522343

[bb9] Sheldrick, G. M. (2007). *SADABS* Bruker AXS Inc., Madison, Wisconsin, USA.

[bb10] Sheldrick, G. M. (2008). *Acta Cryst.* A**64**, 112–122. 10.1107/S0108767307043930 18156677

[bb11] Souza, M. V. N. de, Pinheiro, A. C., Tiekink, E. R. T., Wardell, S. M. S. V. & Wardell, J. L. (2010). *Acta Cryst.* E**66**, o3253–o3254. 10.1107/S1600536810047720 PMC3011551 21589538

[bb12] Souza, M. V. N. de, Pinheiro, A. C., Tiekink, E. R. T., Wardell, S. M. S. V. & Wardell, J. L. (2011). *Acta Cryst.* E**67**, o1868–o1869. 10.1107/S1600536811024895 PMC3151956 21837231

[bb13] Tiekink, E. R. T., Souza, M. V. N. de, Pinheiro, A. C., Wardell, S. M. S. V. & Wardell, J. L. (2011). *Acta Cryst.* E**67**, o1866–o1867. 10.1107/S1600536811025128 PMC3151747 21837230

[bb14] Wardell, J. L., Souza, M. V. N. de, Pinheiro, A. C., Tiekink, E. R. T. & Wardell, S. M. S. V. (2011). *Acta Cryst.* E**67**, o1888–o1889. 10.1107/S1600536811025293 PMC3212283 22090940

[bb15] Westrip, S. P. (2010). *J. Appl. Cryst.* **43**, 920–925.

[bb16] Yakura, T., Yoshimoto, Y., Ishida, C. & Mabuchi, S. (2007). *Tetrahedron*, **63**, 4429–4438.

